# Preparation of graphene oxide–stabilized Pickering emulsion adjuvant for Pgp3 recombinant vaccine and enhanced immunoprotection against *Chlamydia Trachomatis* infection

**DOI:** 10.3389/fimmu.2023.1148253

**Published:** 2023-04-18

**Authors:** Lanhua Zhao, Mingyi Shu, Hongliang Chen, Keliang Shi, Zhongyu Li

**Affiliations:** ^1^ Institute of Pathogenic Biology, Hengyang Medical College, Hunan Provincial Key Laboratory for Special Pathogens Prevention and Control, Hunan Province Cooperative Innovation Center for Molecular Target New Drug Study, University of South China, Hengyang, Hunan, China; ^2^ ILaboratory Department of Chenzhou First People's Hospital, Chenzhou, Hunan, China; ^3^ Institute of Pathogenic Biology, Hengyang Medical College, Hunan Provincial Key Laboratory for Special Pathogens Prevention and Control, The School of Nursing, University of South China, Hengyang, Hunan, China

**Keywords:** graphene oxide, Pickering emulsion, adjuvant, *Chlamydia trachomatis*, immunoprotection

## Abstract

**Background:**

Traditional emulsion adjuvants are limited in clinical application because of their surfactant dependence. Graphene oxide (GO) has unique amphiphilic properties and therefore has potential to be used as a surfactant substitute to stabilize Pickering emulsions.

**Methods:**

In this study, GO–stabilized Pickering emulsion (GPE) was prepared and used as an adjuvant to facilitate an enhanced immune response to the *Chlamydia trachomatis* (*Ct*) Pgp3 recombinant vaccine. Firstly, GPE was prepared by optimizing the sonication conditions, pH, salinity, GO concentration, and water/oil ratio. GPE with small-size droplets was characterized and chosen as the candidate. Subsequently, controlled-release antigen delivery by GPE was explored. Cellular uptake behaviors, M1 polarization, and cytokine stimulation by GPE + Pgp3 was considered in terms of the production of macrophages. Finally, GPE’s adjuvant effect was evaluated by vaccination with Pgp3 recombinant in BALB/c mouse models.

**Results:**

GPE with the smallest droplet sizes was prepared by sonication under 163 W for 2 min at 1 mg/mL GO in natural salinity with a pH of 2 when the water/oil ratio was 10:1 (w/w). The optimized average GPE droplet size was 1.8 μm and the zeta potential was –25.0 ± 1.3 mv. GPE delivered antigens by adsorption onto the droplet surface, demonstrating the controlled release of antigens both *in vitro* and *in vivo*. In addition, GPE promoted antigen uptake, which stimulated proinflammatory tumor necrosis factor alpha (TNF-α), enhancing the M1 polarization of macrophages *in vitro*. Macrophage recruitment was also significantly promoted by GPE at the injection site. In the GPE + Pgp3 treatment group, higher levels of immunoglobin (IgG), immunoglobin G1 (IgG1), immunoglobin G2a (IgG2a) sera, and immunoglobin A (IgA) were detected in vaginal fluid, and higher levels of IFN-γ and IL-2 secretion were stimulated, than in the Pgp3 group, showing a significant type 1 T helper (Th1)-type cellular immune response. *Chlamydia muridarum* challenging showed that GPE enhanced Pgp3’s immunoprotection through its advanced clearance of bacterial burden and alleviation of chronic pathological damage in the genital tract.

**Conclusion:**

This study enabled the rational design of small-size GPE, shedding light on antigen adsorption and control release, macrophage uptake, polarization and recruitment, which enhanced augmented humoral and cellular immunity and ameliorated chlamydial-induced tissue damage in the genital tract.

## Introduction

An adjuvant is an important substance that is used to reduce the required vaccine dosages and enhance vaccine effects. Emulsions are the most widely used type of adjuvant after aluminium adjuvants. Emulsion adjuvants are advantageous because they enhance and regulate immunity, extend immune protection duration, possess excellent physical and chemical properties, and are simple to prepare (1, 2). Several nanoemulsions were screened and optimized in our preliminary study, and animal vaccinations demonstrated greater immunoenhancement effects than Freund’s complete adjuvant (3). However, the surfactant dependence of traditional emulsions is associated with biosafety and stability issues that are yet to be fully addressed (4).

Pickering emulsion refers to a special oil–water system that is stabilized by solid particles. Biocompatible particles such as chitosan, silica, and/or poly(lactic-co-glycolic acid) (PLGA) are used to design Pickering emulsions, which are applied in the drug, adjuvant, and food fields (5, 6). Solid particles are irreversibly adsorbed at the oil–water interface to form a firm interface and avoid droplet coalescence. This ensures their systematic stability and insusceptibility to *in vivo* pH, salinity, or temperature. In addition, a systematic combination of emulsion droplet structure and solid particles may result in double immune enhancement. For example, a Pickering emulsion based on PLGA is more efficient than traditional surfactant-stabilized emulsions owing to its activation of antigen-presenting cells (APCs) and enhanced antigen recruitment, both of which stimulate a highly effective humoral and cellular immune response (7). Therefore, a well-designed Pickering emulsion may solve the safety and stability problems of traditional emulsions and exert excellent adjuvant effects (8, 9).

The properties of solid particles determine the formation and function of Pickering emulsions. Graphene oxide (GO) has emerged in biomedical applications because of its stable physical and chemical properties, easy synthesis, biocompatibility, and modifiability (10). GO is a potential solid particle that may stabilize emulsions because of its unique amphiphilic properties (11, 12). Moreover, the abundant oxygen-containing functional groups on its surface cause the sheets to repel each other and avoid droplet coalescence (13, 14). GO also enhances immune responses if properly functionalized. For example, carnosine-modified GO served as an adjuvant for ovalbumin (OVA) (15) and polyethylene glycol (PEG)-functionalized GO stimulated a robust response when used in combination with the *Helicobacter pylori* urease B subunit vaccine (16, 17). These studies provided strong evidence for the immune-enhancing effect of GO, but the complex functional modifications make its widespread application as an adjuvant impractical because of the high cost associated with its large-scale preparation. A GO-stabilized Pickering emulsion (GPE) prepared by simple sonication self-assembly may therefore represent a novel strategy to enhance its adjuvant effect and batch production.

*Chlamydia trachomatis* is responsible for ocular or genital infections such as blindness, urethritis, cervicitis, and salpingitis, with over 130 million new cases being recorded every year according to the most recent estimates from the World Health Organization (18). *C. trachomatis* causes considerable morbidities and exerts a huge socioeconomic burden on human healthcare (19). Unfortunately, there is no clinical vaccine for humans (20). Pgp3 is an important virulence protein encoded by the cryptic plasmid (21–24). Our previous studies confirmed that Pgp3 was a conformation-dependent immunodominant antigen, which was an expected vaccine candidate (25). However, immunoprotection is not enough to combat *C*. *trachomatis* infection because it results in chronic infection of the reproductive tract and tubal lesions. Therefore, treatment with a highly effective adjuvant is a common strategy.

This study aimed to design small-sized GPE droplets by optimizing the sonication conditions, pH, salinity, GO concentration, and water/oil ratio. Subsequently, interactions between GPE and Pgp3 were explored through sustained release and adsorption rate. Cellular uptake, cytokine production, and the M1/M2 polarization of macrophages stimulated by GPE + Pgp3 are considered to be indicative of the *in vitro* immunoenhancement. We seek to determine GPE’s adjuvant effect by establishing the innate immunity, such as the recruitment of APCs at the injection site, and adaptive immunity, such as antibody and cytokine production. We hope that this provides new ideas for the design of Pickering emulsion and its application on *C. trachomatis* Pgp3 recombinant, which results in a reduced genital chlamydial load in the lower genital tract and less inflammation pathology in the upper reproductive tract, therefore providing an efficient adjuvant strategy against *C*. *trachomatis* infection.

## Materials and methods

### Materials

GO dispersion was purchased from Xianfeng Nano Technology Co., Ltd, Nanjing, China. Squalene was from Macklin Biochemical Co., Ltd, Shanghai, China. Pgp3 was purified by affinity resin with the glutathione S-transferases (GST) tag removed. ELISA MAX™ Deluxe mouse kits were purchased from Biolegend USA. Fluorescein isothiocyanate (FITC) goat anti-mouse IgG was purchased from Absin Bioscience Inc and Cy7-BSA was purchased from Xian Qiyue Biology. Fluorescently labelled antibody CD45-PE, CD11b-APC, CD11b-PerCP Cy5.5, PE-CD86, CD206-FITC, F4/80-FITC, and F4/80-PerCP Cy5.5 were purchased from Biolegend. Specific pathogen-free BALB/c mice (SYXK-2020-0002) were bought from SJA Laboratory Animal Co., Ltd, Hunan, China. All animal experiments were carried out with the authorization of the animal ethics committee of the University of South China. All procedures were performed in accordance with the Laboratory Animal Management Regulations in China.

### Preparation and optimization of GPE

GO (1–6 layers, 500–5,000 nm, 10 mg/mL) was adopted as a stabilizer for the preparation of Pickering emulsion and was diluted 10-fold with double-distilled water. The initial concentration of the GO dispersion was 10 mg/mL, which was diluted to the given concentration with double-distilled water or various phosphate buffered saline (PBS) solutions (dilutions were one-, two-, five-, and 10-fold). The pH of the GO dispersion was adjusted using 1 M sodium hydroxide and concentrated hydrochloric acid. GPE was formed by mixing the GO dispersion with squalene at water/oil ratios of 10:4, 10:2, and 10:1 (w/w), shaken thoroughly for 30 seconds, and then quickly dispersed on ice by sonication at powers of 65, 163, 326, or 652 W, for 1, 2, 5, or 10 min. The optimized GPE was stored at room temperature for 60 days and observed every 30 days to assess its long-term storage stability.

### Characterization of GPE

GPE was stored overnight, then appropriately diluted with double-distilled water for micro-observation using a Nikon Ts2r inverted microscope, with three random images of each emulsion taken for droplet size analysis. Zeta potential was measured using a Malvern Zetasizer Nano ZS90. The average droplet sizes were analyzed by Nanomeasure1.2 software to construct a size distribution diagram. Droplet size data were analyzed by Graphpad Prism 7.0 to construct a line chart displaying change tendency.

### Controlled release of GPE *in vitro* and *in vivo*

Because it was difficult to efficiently label Pgp3 with fluorescent dye, another model antigen, fluorescein labeled bovine serum albumin (FITC-BSA), was used instead in the interaction mode experiment. FITC-BSA was mixed with GPE at room temperature for 4 h (500 μL GPE with 250 μg BSA), then observed in both bright and dark fields to determine if antigens were on the surface of GPE or inside the droplet.

The adsorption rate of Pgp3 was measured by centrifugation at 3,000 g for 20 min in an ultrafiltration tube to collect the free protein and calculate the adsorption rate.

The controlled release of Pgp3 was detected with PBS as a control. The GPE + Pgp3 mixtures were pipetted into a micro tube dialyzer™ (50 kD MW) and the tube was placed into 2 mL PBS for dialysis. A 50-μL extracalytic fluid sample was taken every 24 h to detect the protein concentration. Meanwhile, 50 μL PBS was added to the dialysate to maintain the external liquid volume.

Cy7-BSA was used for *in vivo* imaging because it was difficult to label Pgp3 with high efficiency. PBS+Cy7-BSA or GPE+Cy7-BSA (100 μL GPE containing 50 μg Cy7-BSA) were subcutaneously injected into the abdomens of BALB/c mice. Each group contained three 7-week-old female mice. Mice were anesthetized with 0.1 mL of 5% chloral hydrate by intraperitoneal injection. Images were taken at 2, 12, 24, 36, and 48 h by Vilber Fusion FX Spectra, with the excitation wavelength set at 740 nm. The fluorescence intensity at the injection site was analyzed to compare the reservoir effect of antigen.

### Macrophage uptake, polarization, and cytokine secretion

Cellular uptake involved incubating the Raw 264.7 cells (2 × 10^6^ cells/mL in 24-well plates) in Dulbecco’s modified Eagle medium (DMEM) with Pgp3 (10 μg/mL), GPE (20 μL/mL), or GPE+Pgp3 (20 μL GPE+10 μg BSA), and subsequently mixing by pipetting up and down several times and adsorbing at room temperature for 4 h. PBS was added as the control, and this process was performed for each group in triplicate. The cells were cultured at 37° in 5% CO_2_ for 4 h. DMEM was removed and the cells were washed with PBS three times to remove dissociative Pgp3 or GPE. Cells were fixed using IC-fixation buffer for 40 min and permeabilized twice using intracellular fixation and permeabilization buffer for 5 min, after which anti-mouse Pgp3 serum was diluted (at a ratio of 1:1,000) and incubated with Raw 264.7 cells at 37° for 1 h before being washed with PBS three times. Finally, FITC anti-mouse IgG was diluted (at a ratio of 1:5,000), incubated with cells for 1 h and washed three times before flow cytometry detection or in-plate microscope observation.

The same incubation procedure was performed for macrophage polarization and cytokine secretion, except that in this instance the incubation time was 48 h. The supernatant was collected for cytokine detection [tumor necrosis factor alpha (TNF-α), interleukin-1 beta (IL-1β), and interleukin-10 (IL-10)] using an enzyme-linked immunosorbent assay (ELISA) kit. Meanwhile, cells were collected for fluorescent antibody labeling. 1 μL of PerCP/Cyanine5.5 anti-mouse F4/80 antibody, PE anti-mouse CD86 antibody, or APC anti-mouse CD11b antibody, was added to 100 μL PBS and incubated with the cells for 20 min on ice. Washing with PBS and fixation and permeabilization were carried out before FITC anti-mouse CD206 antibody incubation. Finally, the cells were washed and suspended in 300 μL PBS for flow cytometry detection.

### Macrophage recruitment at the injected site

A total of 12 female 7-week-old BALB/c mice were divided into three groups, with each group containing four mice. For the PBS group, each mouse was immunized with 100 μL PBS. For the Pgp3 group, each mouse was immunized with 50 μg Pgp3 dissolved in 100 μL PBS. For the GPE + Pgp3 group, each mouse was immunized with a total volume of 100 μL GPE containing 50 μg Pgp3. Subcutaneous tissues from the injection site were collected 24 h post injection, then were dissected and minced before being co-incubated with digestion buffer for 40 min at 37°. Each tissue sample was digested by 3 mL DMEM with 1 mg/mL type VI collagenase D (Gibco, Thermo fisher scientific, USA), 0.25 mg/mL DNase I (Ruibio, China), and 10% fetal bovine serum (FBS). Thereafter, the digested tissue was filtered through a 70-μm cell strainer to acquire a single cell suspension, which was used for fluorescent labeling by F4/80-FITC, CD45-PE, and CD11b-PerCP-Cy5.5 and detected by BD FACS Calibur flow cytometry. The expression of surface markers was assessed using FlowJo V10.0 software.

### Animal immunization

A total of 30 female 5-week-old BALB/c mice were randomly divided into three groups (10 mice per group) and immunized with PBS, Pgp3, or GPE + Pgp3 *via* subcutaneous injection at the abdominal site (100 μL GPE with 50 μg Pgp3 per mouse). All mice were immunized at weeks 0, 2, and 4. Moreover, different adjuvant vaccination doses (original emulsions and the emulsion diluted five times by PBS) were investigated. Sera were collected from the caudal vein 1 day before each immunization, and the sera samples were stored at −20°. Vaginal fluid was collected by rotating vaginal swabs 10 times clockwise and counterclockwise in mice vaginas three times 2 weeks post final immunization. They were then placed in 1 mL PBS and thoroughly vortexed for 2 min. The washed PBS was then used as vaginal lavage fluid for IgG, IgG1, IgG2a, and IgA detection.

### ELISA for detection of IgG antibody

An ELISA was conducted to quantitatively calculate IgG, IgG1, and IgG2a levels to detect the Pgp3 specific antibody titer. A total of 10 μg of recombinant Pgp3 was added to each well and incubated overnight at 4°C. After blocking using 5% skimmed milk in PBST for 2 h, 50 μL serum sample was added and incubated at 37° for 1 hour. Each sample was subjected to a series of double dilutions, with dilution ratios ranging from 1:10,000 to 1:1,000,000 according to the immunization week. After washing four times with PBS and 0.05% Tween 20, 100 μL diluted (1:5,000) horseradish peroxidase (HRP)-labelled goat anti-mouse antibody was added and incubated for 1 hour. The chromogenic reaction involved the addition of 100 μL 3,3’,5,5’-tetramethylbenzidine solution, followed by incubation at 37° for 30 min. The reaction was stopped by the addition of 50 μL 2 M sulfuric acid (H_2_SO_4_). Finally, absorbance values were read at 450 nm within 10 min. Antibody titers were judged by the maximum dilution resulting in an optical density (OD_450_) value greater than twice the mean absorbance of the negative serum. For vaginal fluid samples, the indirect ELISA procedure was similar to serum IgG detection, except that 100 μL vaginal lavage fluid was added rather than a series of double dilutions, and HRP-labeled goat anti-mouse IgA antibody was diluted at a ratio of 1:500 and used as the secondary antibody. The resulting OD_450_ value was recorded for statistical analysis.

### Cytokine detection

Mice were sacrificed 2 weeks after the final immunization (*n* = 5). Spleen cells were collected and washed with Hank’s solution, treated with 5 mL red blood cell lysis buffer for 5 min. Subsequently, 5 mL DMEM was added to stop the lysis and centrifuged at 700 g for 5 min. The cells were resuspended in DMEM supplemented with 10% FBS and 1% penicillin–streptomycin–L-glutamine. Thereafter, 1 × 10^6^ cells/mL in 24-well plates were co-cultured with 10 μg Pgp3 for 48 h and the cytokine levels in the supernatant [IL-4, interferon gamma (IFN-γ), IL-2, and IL-10] were determined by ELISA assays in accordance with the kit instructions.

### *Chlamydia muridarum* challenging

*Chlamydia muridarum* challenging was carried out for three groups, namely the PBS, Pgp3, and GPE + Pgp3 groups, 2 weeks after the final immunization. Seven days prior to *C. muridarum* challenging, each mouse was injected with 2.5 mg medroxyprogesterone to synchronize the estrus cycle and then was intravaginally challenged with 1 × 10^5^ infectious units (IFU) of *C. muridarum* in 15 μL sucrose-phospho-glutamine buffer (SPG) through the vaginal posterior fornix 2 weeks post final immunization (*n* = 5). Vaginal swabs were collected 5 days post challenge for indirect ELISA test on secretory immunoglobin A (sIgA).

Vaginal swabs were collected every 7 or 3 days until *C. muridarum* was completely cleared, to plot the *C. muridarum* burden–time curve. Mice were sacrificed 60 days post *C. muridarum* infection to evaluate urogenital tract tissue pathology, and the intact reproductive tracts of mice were stripped and photographed. The oviduct hydrosalpinx was visually scored based on their dilation size using the scoring system described in a previous study (26), and then were placed in 4% paraformaldehyde for pathological sections observation by hematoxylin–eosin staining.

### Statistical analysis

All results were displayed as means ± SD from at least three independent experiments unless otherwise indicated. Statistical differences were determined by using a one-way ANOVA test unless otherwise specified, and a *t*-test was adopted for a two-group comparison. Analyses were performed using GraphPad Prism 7.0 (San Diego, CA, USA). All analyses were shown compared to the control, and the significance of the differences was indicated as * *p *< 0.05, ** *p* < 0.01, or *** *p* < 0.001.

## Results

### Optimization of GPE by different sonication power and time

Sonication is a simple method that uses high energy to create droplets that are smaller than those generated by other homogenizing methods such as vortexing. The effects of sonication power and time on the morphologies of GPEs are shown in the Supplementary Material. Increasing sonication power reduced the GO utilization rate and emulsification efficiency at 1 mg/mL GO, because the upper emulsion volume slightly decreased while the lower layer gradually became darker (**Supplementary Figure 1A**). The average droplet size and size distribution slightly changed when the sonication power increased from 65 W to 652 W (**Supplementary Figures 1B-J**). A similar phenomenon was observed at 2 mg/mL GO (**Supplementary Figure 2**). Thus, a sonication power of 163 W was used in the follow-up optimization.

The effects of sonication time on GPE morphology are shown in **Supplementary Figures 3**, **4**. The upper emulsion volume decreased, and the lower layer gradually became darker as the sonication time was prolonged from 1 to 10 min at 1 mg/mL GO (**Supplementary Figure 3A**). The average droplet size and size distribution did not significantly change with increasing sonication time (**Supplementary Figure 3B-J**). A similar phenomenon was observed at 2 mg/mL GO (**Supplementary Figure 4**), and we chose 2 min as the uniform ultrasound time in the following optimizations.

### Optimization of GPE by different GO concentrations

**Figure 1:**
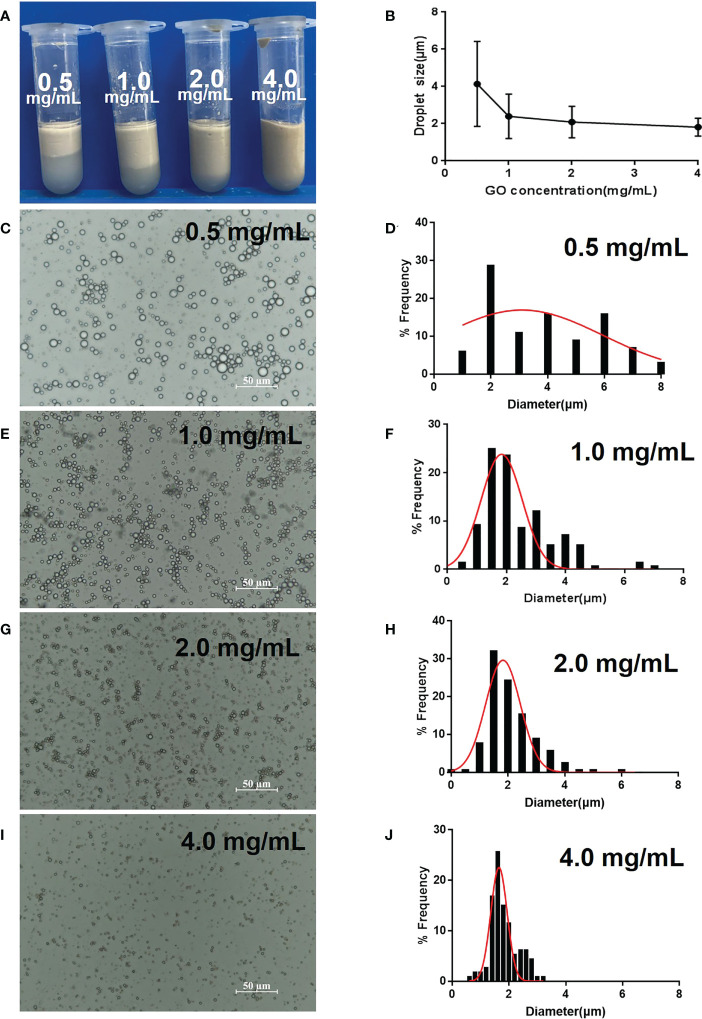
Optimization of GPE based on different GO concentrations. **(A)** Appearance of GPEs prepared using different GO concentrations; **(B)** the average droplet size as a function of GO concentration; **(C, E, G, I)** optical micrographs and **(D, F, H, J)** droplet size distributions of the GPEs prepared using different GO concentrations. Preparation conditions: water/oil ratio 10:2, sonication at 163 W for 2 min, with squalene as the oil phase under natural salinity and pH conditions. GO, graphene oxide; GPE, GO-stabilized Pickering emulsion.

The emulsion volume significantly increased as the GO concentration increased from 0.5 to 4.0 mg/mL and there was almost no stratification at 4.0 mg/mL (**Figure 1A**). In contrast, the droplet size decreased and became uniform with an increased GO concentration (**Figure 1B–J**). The GPE droplet size (2.2 μm at 1.0 mg/mL GO) was much smaller than that of GO-benzyl chloride (15 μm) at the same GO concentration (**Figures 1E, F**). An increase in GO concentration slightly decreased the droplet size, which stabilized at approximately 2 μm (**Figures 1H, J**). As a result, a GO concentration of 1.0 mg/mL was used in the following optimization and animal experiments because only a slight increase in droplet size was observed above this concentration.

### Optimization of GPE by different pH value and salinity

The water phase color became darker, and the emulsion volume became smaller with increasing pH, indicating that an acidic environment was conducive to emulsion formation, whereas neutral and alkaline environments reduced GO utilization (**Supplementary Figure 5A**). We observed that pH had no obvious influence on the average droplet size, although it widened the size distribution (**Supplementary Figure 5B**). Large droplets appeared when the pH increased to 5 (**Supplementary Figures 5G, H**), which further increased in size at pH values of 7 and 10 (**Supplementary Figures 5I, K**). Overall analysis of the appearance and droplet size indicated that a pH of 2–3 facilitated emulsion formation for small and uniform droplets.

**Figure 2:**
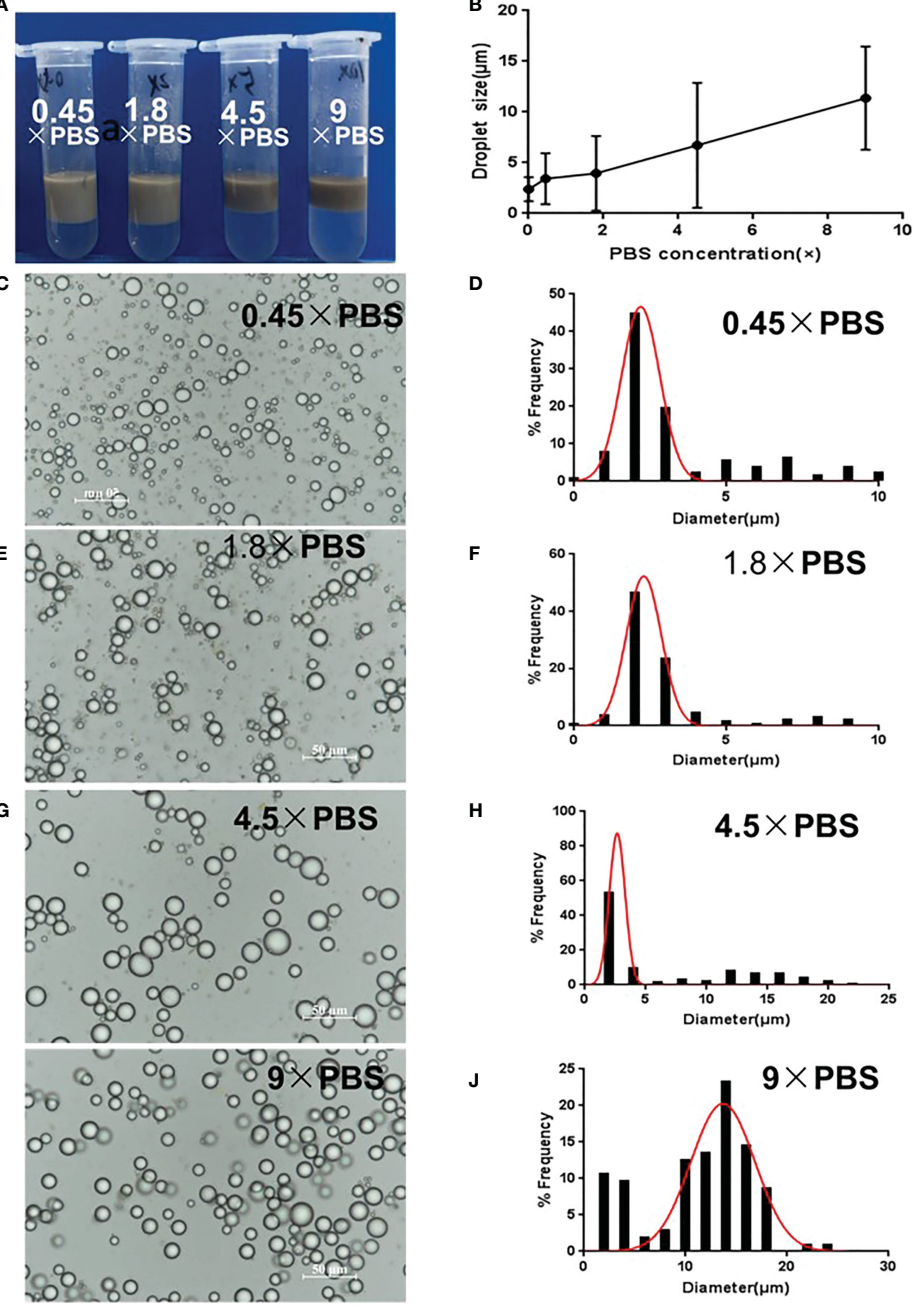
Optimization of GPE based on different salinities. **(A)** Appearance of GPEs prepared at different salinities; **(B)** the average droplet size as a function of salinity; **(C)** GO dispersions diluted to 1 mg/mL with different concentrations of PBS; **(D)** GO dispersions with different salinities when mixed with squalene; **(C, E, G, I)** optical micrographs and **(D, F, H, J)** droplet size distributions of the GPEs prepared at different salinities. Preparation conditions: water/oil ratio 10:2, GO concentration 1.0 mg/mL, sonication at 163 W for 2 min. GO, graphene oxide; GPE, GO-stabilized Pickering emulsion; PBS, phosphate-buffered solution.

Dilution of GO dispersion by variable PBS concentrations resulted in different emulsion formations from that diluted by water (**Figure 2**). GO dispersion in 0.45 × PBS or 1.8 × PBS slightly gathered and hardly entered into the oil phase. However, increasing PBS concentration promoted the stacking and aggregation of GO sheets, causing floccule formation and precipitation, which completely entered the oil phase after mixing with squalene. This was due to salt ions reducing the repulsive force between GO sheets, which caused accumulation and disequilibrium, and an inclination to enter the oil phase. Consequently, with increasing salinity, the emulsion gradually became darker brown in color, whereas the lower water layer became colorless and transparent (**Figure 2A**), indicating that GO was heavily involved in emulsion formation. The average droplet size increased, and the distribution widened with increasing salinity. The average size was approximately 3–4 μm in 0.45 × PBS and 1.8 × PBS (**Figures 2C–F**). The droplet size was 5–15 μm, with decreased uniformity at 4.5 × PBS and 9 × PBS (**Figures 2B, G–J**). The optimal GO dispersion that stabilized the water–oil interface and produced smaller uniform droplets was formed at lower ionic concentrations. Consequently, natural GO without PBS and at a pH of 2–3 was suitable for emulsion formation.

### Optimization of GPE by different water/oil ratio

**Figure 3:**
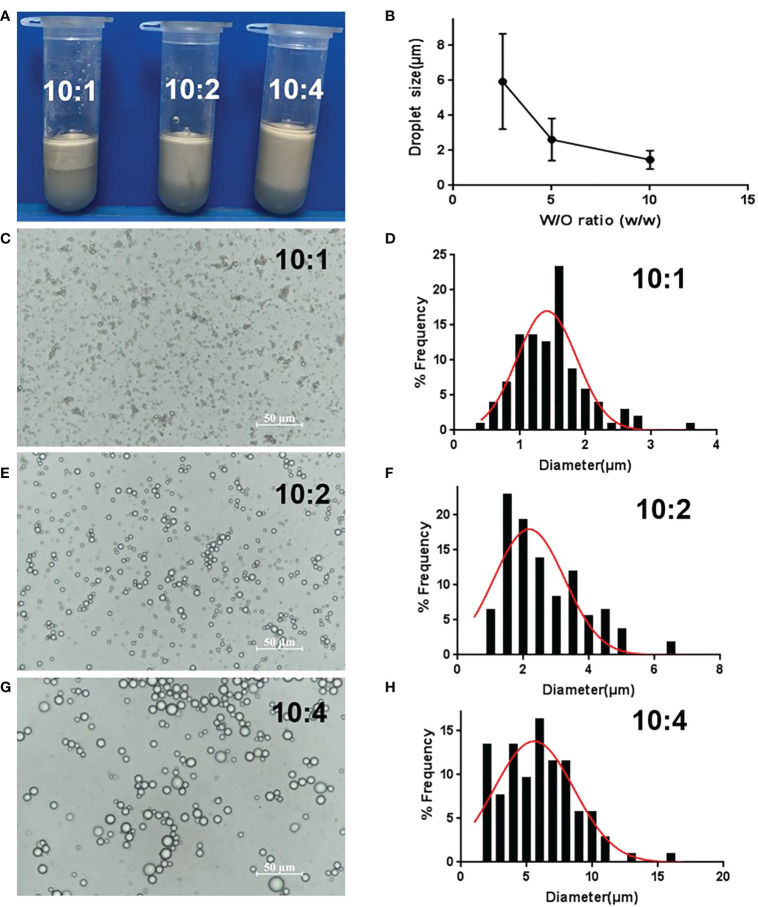
Optimization of GPE based on different water/oil ratios. **(A)** Appearance of GPEs prepared at different water/oil ratios; **(B)** the average droplet size as a function of the water/oil ratio **(C, E, G)** optical micrographs and **(D, F, H)** droplet size distributions of the GPEs prepared at different water/oil ratios. GO concentration of 1.0 mg/mL, sonication at 163 W for 2 min, natural salinity, and pH of 2–3. GO, graphene oxide; GPE, GO-stabilized Pickering emulsion.

The volume of the upper layer emulsion increased as the water/oil ratio increased, because the oil phase lowered the emulsion’s density (**Figure 3A**). As for droplet size, the droplet diameter increased from 1.8 μm to 6 μm as the water/oil ratio changed from 10:1 to 10:4 (**Figures 3C–H**), that is the droplet size increased as the oil ratio increased (**Figure 3B**). In fact, more oil phase meant a decrease in GO concentration. Therefore, less oil phase and an increased number of GO particles were conducive to the formation of small-sized droplets in a certain range. Considering the cost of GO and squalene, 1 mg/mL GO and a water/oil ratio of 10:1 was chosen as the optimized conditions.

Based on the above results, sonication conditions had relatively little influence on GPE, whereas pH, salinity, GO concentration, and the water/oil ratio were key factors determining droplet size. Small GPE droplets were prepared using 1 mg/mL GO and a water/oil ratio of 10:1, with no PBS, pH = 2, and sonication power at 163 W for 2 min.

### Characterization of GPE

Droplet size and zeta potential were measured since they greatly affect both Pickering emulsion stability and its biological application. Microscopic observations showed that GO nanosheets were concentrated at the oil–water interface, which is a typical characteristic of Pickering emulsion (**Supplementary Figure 6**). GPE was stable at room temperature within 60 days of observation because the droplet size and appearance were relatively unchanged (**Supplementary Figure 7**). The average droplet size of GPEs was 1.8 μm and was negatively charged at –25.0 ± 1.3 mv, and GPE absorbed Pgp3 at a rate of 80.2%.

To fit with applications in the near-neutral pH environment in animals and cells, GPE was centrifuged at a rate of 3,000 g for 10 min. Subsequently, the lower aqueous phase was discarded and resuspended with an equal volume of PBS. Microscopical observation was carried out overnight post PBS resuspension. Average droplet size analysis showed that centrifugation and PBS treatment did not change the droplet morphology or size (**Supplementary Figure 8**). The remoulded GPE was adopted in the following cell and animal experiments.

### GPE controlled-released antigen *in vitro* and served as an antigen reservoir *in vivo*

**Figure 4:**
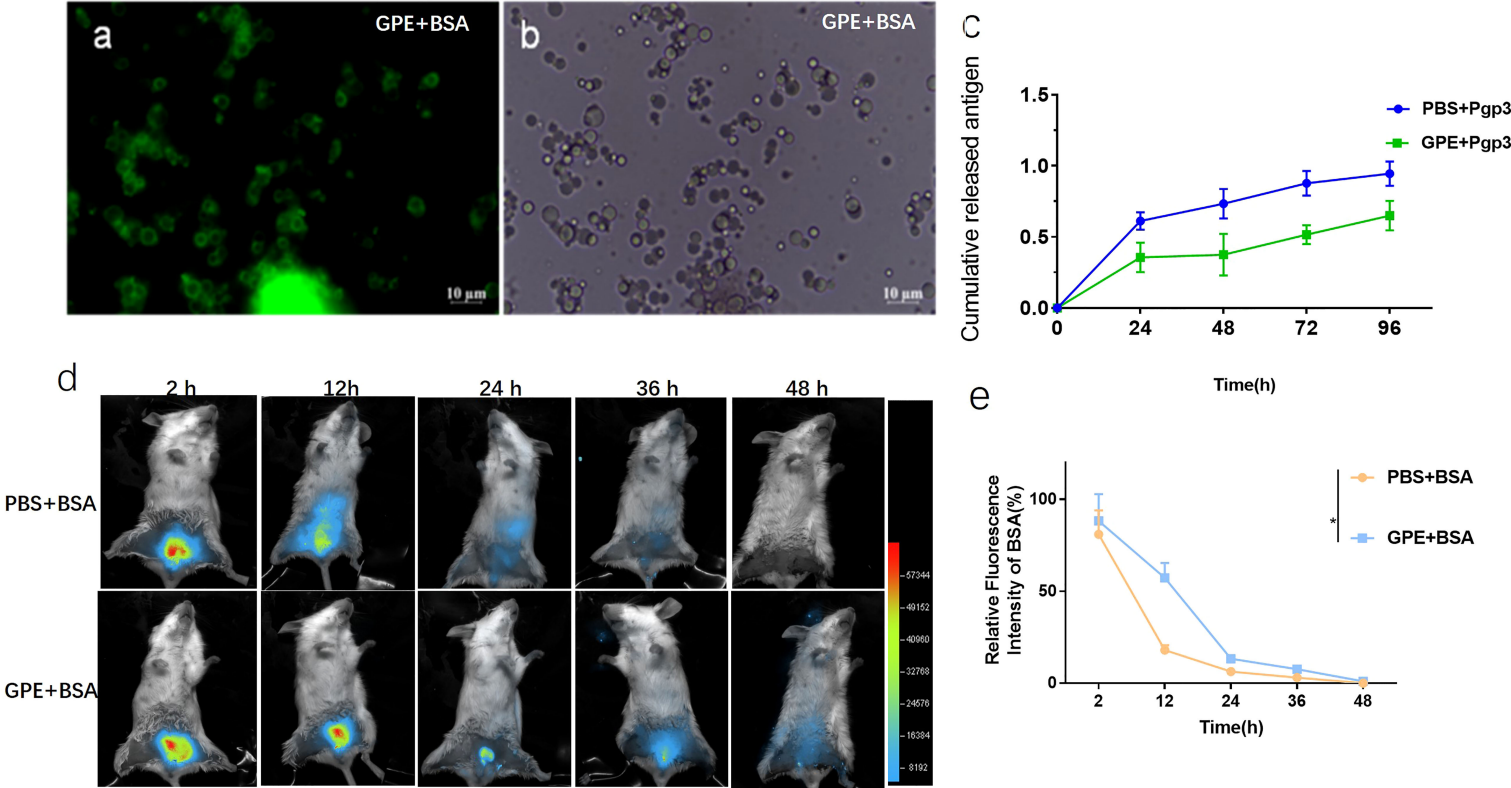
Optical micrograph of GPE after protein absorption. **(A)** GPE adsorbed FITC-BSA observed in bright field; **(B)** GPE adsorbed FITC-BSA observed in dark field; **(C)** antigen-controlled release of GPE + Pgp3; **(D)** fluorescence intensity distribution at the injected site of representative mice; **(E)** fluorescence intensity distribution at the injected site of representative mice, *n* = 3; all comparisons were done by two-way ANOVA here. FITC-BSA, fluorescein labeled bovine serum albumin; GO, graphene oxide; GPE, GO-stabilized Pickering emulsion.

FITC-labelled BSA was mainly distributed at the water–oil interface rather than being encapsulated in the droplets (**Figures 4A, B**). As BSA and Pgp3 (pI = 4.7) were positively charged in the PBS, GPE adsorbed the two proteins because of its negative zeta potential. In terms of Pgp3 release, GPE showed a slower release than PBS over 96 h (**Figure 4C**). *In vivo* fluorescent intensity at the injected sites suggested that the antigen accumulation of GPE was higher than that of PBS during a 48-h observation, indicating the occurrence of the reservoir effect at the injection site (**Figures 4D, E**).

### GPE enhanced macrophage uptake, M1 polarization, and cytokine secretion

**Figure 5:**
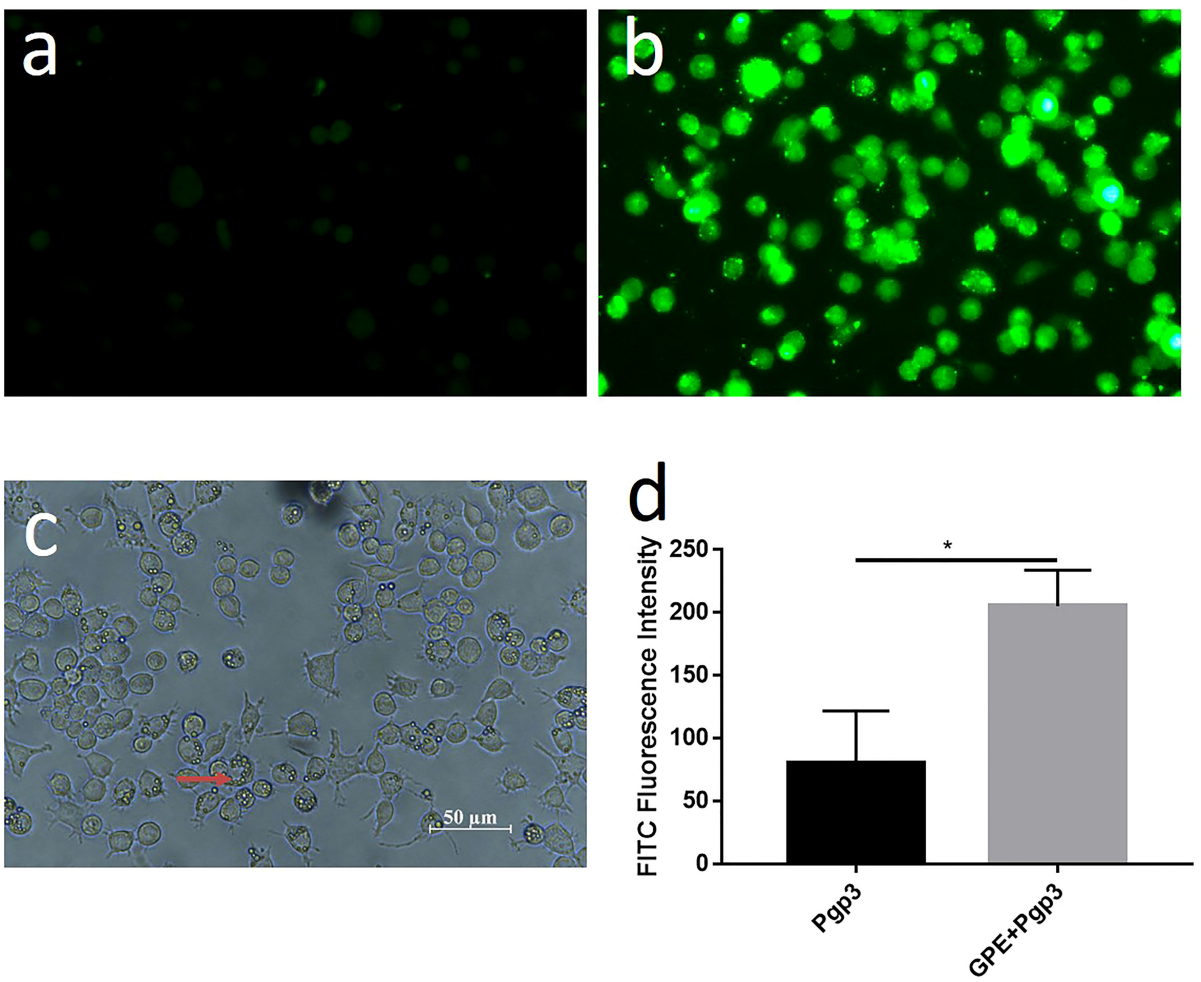
Cellular uptake of GPE and Pgp3 (micro image of Pgp3 uptake post incubation 4 h with Raw 264.7 cells. **(A)** Pgp3; **(B)** GPE+ Pgp3 in dark field; **(C)** GPE+Pgp3 in bright field; **(D)** statistical analysis of FITC fluorescence intensity 4 h post uptake (*n* = 3). FITC, Fluorescein isothiocyanate.

We also explored the uptake of GPE droplets and cytokine production by macrophages. GPE droplets were efficiently taken up by macrophages after co-incubation, and several droplets were phagocytosed into one cell or even filled up the entire cytoplasm. Approximately 50% of macrophages underwent phagocytosis at a concentration of 20 μL/mL, with no obvious cell apoptosis (**Figure 5C**, **Supplementary Figure 9**). The cumulative effect of fluorescence intensity over time indicated that GPE + Pgp3 phagocytosis was more effective than Pgp3 after co-incubation with macrophages (**Figures 5A–D**).

**Figure 6:**
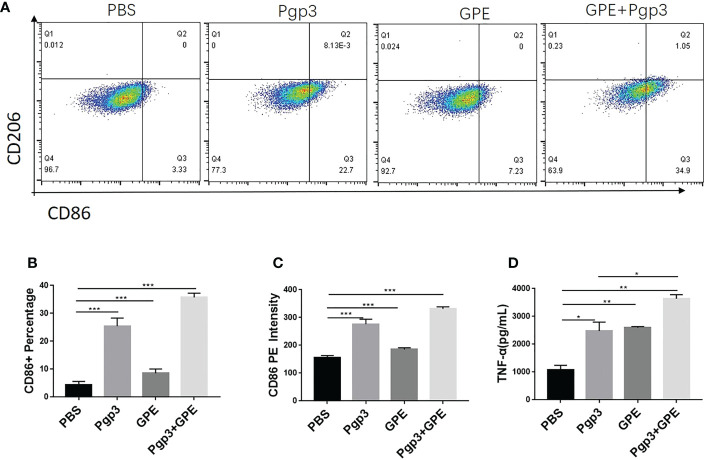
M1/M2 polarization of macrophages. **(A)** Representative flow cytometry dot plot analysis of macrophages treated by GPE and Pgp3; **(B)** statistical analysis flow cytometry dot plot of CD 86^+^ macrophage percentage; **(C)** statistical analysis of CD86-PE fluorescence intensity; **(D)** TNF-α secreted by macrophages incubated for 48 h. (*n* = 3). GPE, GO-stabilized Pickering emulsion; TNF-α, tumor necrosis factor alpha.

In view of the key role that macrophages play in natural immunity, GPE and Pgp3 were co-incubated with Raw 264.7 cells for 24 h at the same concentration as that used for cellular uptake. TNF-α inflammatory cytokine was significantly up-regulated in the supernatant after Pgp3 or GPE treatment, which was two to four times higher than in the PBS-treated group, and the adsorption of Pgp3 and GPE showed a synergistic effect (**Figure 6D**). For macrophage polarization, Pgp3 and GPE induced M1 macrophage activation and showed synergy (**Figure 6A**). A statistical analysis of PE-CD 86 fluorescence intensity and cell percentage showed that the CD11b^+^/F4/80^+^ macrophage expressed two-fold higher levels of the CD86+ marker after treatment with Pgp3 and GPE, respectively, compared with untreated cells (**Figures 6B, C**).

### GPE promoted macrophage recruitment at the injection site

**Figure 7:**
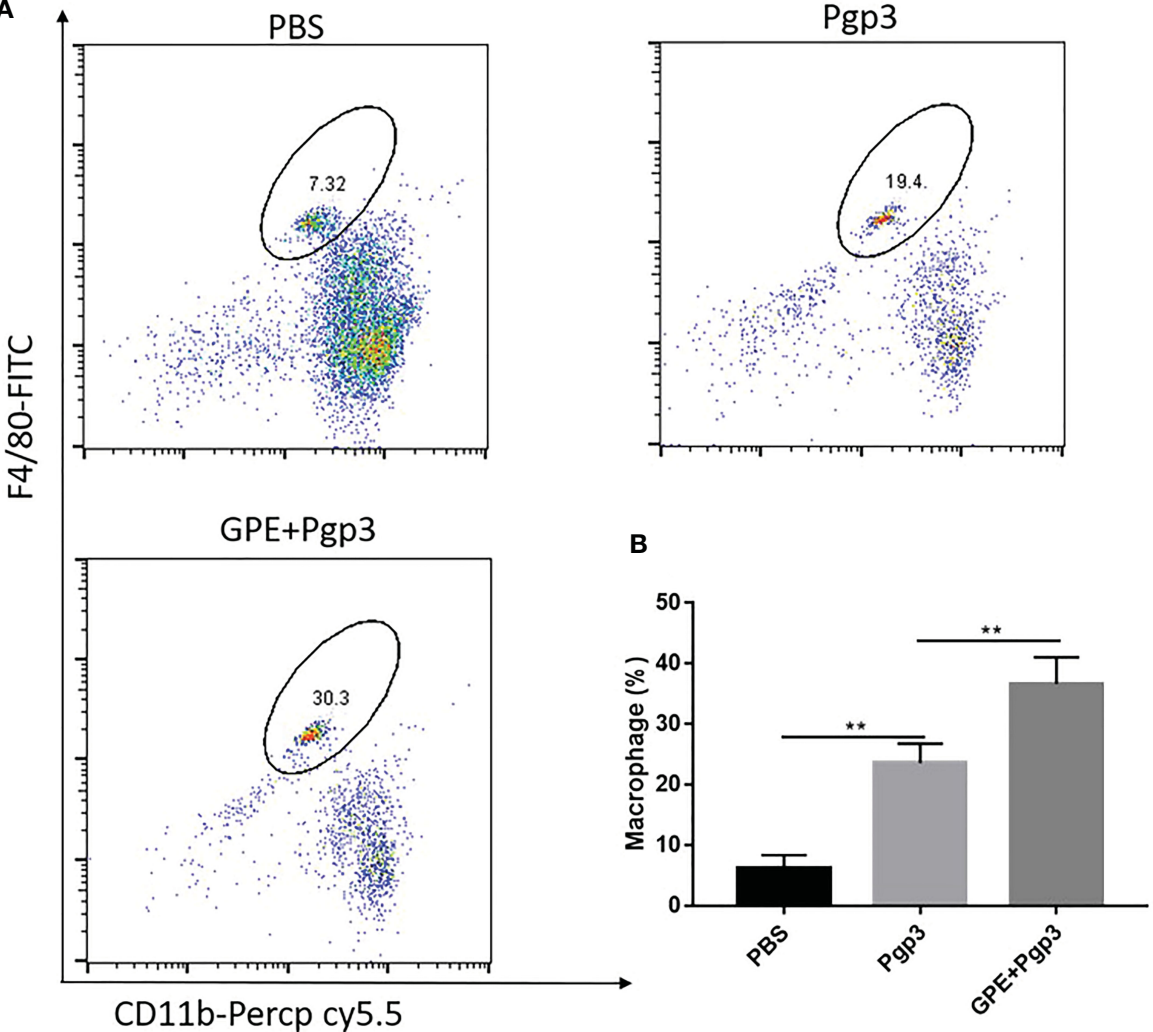
Macrophage recruitment at the injection site. Female BALB/c mice (n = 4) were injected with either NS, Pgp3 (50 μg), or GPE+Pgp3 (50 μg Pgp3 in 0.1 mL GPE). Flow cytometry analysis of CD11b^+^ F4/80^+^ in representative mice **(A)** and statistical comparison **(B)** of macrophages 24 h post injection of GPE. GPE, GO-stabilized Pickering emulsion.

Mice that were injected with Pgp3 showed higher levels of macrophage recruitment at the injection site than those injected with PBS because of antigen stimulation. Importantly, GPE+Pgp3 showed a higher macrophage frequency than the Pgp3 group (*p* < 0.001) (**Figures 7A, B**). Overall, mice injected with GPE + Pgp3 showed efficient recruitment of macrophages at the injection site, and this might play an important role in antigen recognition and presentation, in turn leading to enhanced innate immunity.

### GPE stimulated IgG antibody production

**Figure 8:**
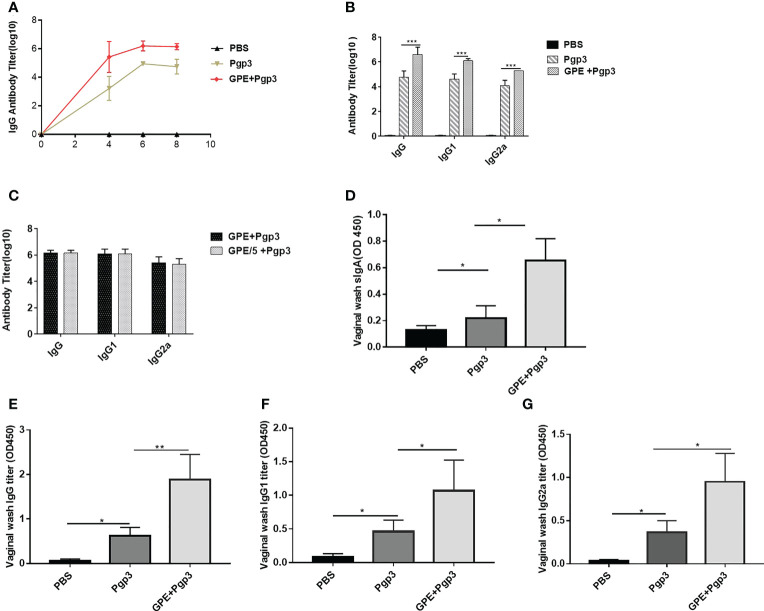
Antibody response post three immunizations. **(A)** Serum IgG titer at 4th, 6th, and 8th week; **(B)** IgG serum and its subtype titers; **(C)** IgG serum and its subtype titer of different GPE dose; sIgA **(D)**, IgG **(E)**, IgG1 **(F)**, IgG2a **(G)** in vaginal fluid, *n* = 5. GPE, GO-stabilized Pickering emulsion.

Indirect ELISA was carried out to detect the antibody levels of each group. The IgG titer increased sharply after the first immunization, with those of the GPE groups being significantly higher than that of the Pgp3 group. In addition, stable growth was observed in the GPE groups after two or three immunizations. Antibody levels in the GPE groups were over tenfold higher than that of the Pgp3 group, indicating an immense potential for higher levels of antibody secretion (*p* < 0.001) (**Figure 8A**). The high levels of IgG2a and IgG1 detected confirmed the significantly high antibody levels in the GPE group (**Figure 8B**). In addition, the fivefold dilution of GPE showed no difference in IgG, IgG1, and IgG2a levels compared with the undiluted, indicating that GPE might be an efficient adjuvant, with the ability to elicit a high immune response at even small doses (**Figure 8C**). In addition, mice in the GPE + Pgp3 group were found to have higher Pgp3-specific IgG antibody levels as well as the subtype in the vaginal fluid samples (**Figures 8E–G**). Notably, the sIgA titer in the vaginal fluid was also enhanced by GPE (**Figure 8D**). Because *Chlamydia trachomatis* is a mucosal pathogen, the presence of Pgp3-specific IgG and sIgA antibodies in vaginal fluid proved to be especially beneficial for its clearance when mice were challenged from the genital tract.

### GPE induced Th1- type cytokine production

**Figure 9:**
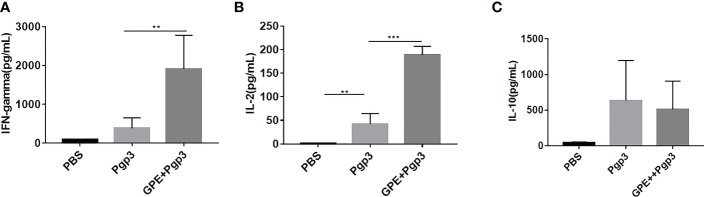
Cytokine **(A)**, IFN-γ **(B)**, IL-2 **(C)**. IL-10 level of splenocyte culture supernatant of BALB/c mice (mice were sacrificed 2 weeks after the final immunization. Spleen cells were stimulated by 10 μg Pgp3 for 48 h to collect the supernatant for an ELISA test (*n *= 5)). ELISA, enzyme-linked immunosorbent assay.

The levels of IFN-γ, IL-4, IL-2, and IL-10 represented the cellular immune response in mice. IFN-γ and IL-2 were characteristic cytokines of the Th1 cellular immune response, whereas IL-4 and IL-10 were characteristic cytokines of the type 2 T helper (Th2) cellular immune response. GPE + Pgp3 stimulated fourfold higher levels of IFN-γ production in mice than Pgp3 (**Figure 9A**), and levels of IL-2 production were also fourfold higher than in the GPE + Pgp3 than the Pgp3 group (**Figure 9B**). There was no difference in IL-10 production among all groups (**Figure 9C**). Neither the Pgp3 nor the GPE + Pgp3 group failed to stimulate IL-4 production. Overall, GPE stimulated a Th1-type cellular immune response, which was similar to most emulsion adjuvants.

### GPE displayed immunoprotection against *C. muridarum* challenging in the mouse model

**Figure 10:**
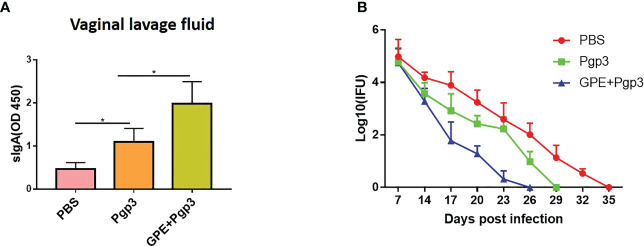
sIgA level in and *Chlamydia muradirum* (*C. muradirum*) clearance curve in vaginal tract post challenging. **(A)** sIgA in vaginal lavage fluid; **(B)** line chart for the **(C)** *muradirum* burden (Log10 IFU) of each group post infection.

There was a significant difference in sIgA levels in the vaginal lavage fluids of the PBS and Pgp3 groups 5 days post *C. muridarum* infection, and the mean OD value of the Pgp3 group was twice as high as that of the PBS control group. Moreover, mice in the GPE+Pgp3 group secreted higher levels of sIgA in vaginal mucosa than those in the Pgp3 group (**Figure 10A**), which indicates that there is a more efficient mucosal defense against *C. muridarum* colonization in the lower genital tract. Bacterial burden was direct evidence for immune protection. Mice in the Pgp3 group showed a shorter clearance time than those in the PBS control group, indicating the superior immunoprotection imparted by the Pgp3 subunit vaccination against *C. muridarum* infection. Those in the GPE + Pgp3 group showed the lowest chlamydia burden in all immunofluorescence tests, as well as a significantly reduced *C. muridarum* clearance time when compared with the Pgp3 or PBS groups (**Figure 10B**). In addition, the area under the *C. muridarum* IFU-days post-infection line chart for the GPE + Pgp3 group was much smaller than those for the Pgp3 and PBS control groups. In addition, 40% of mice in the GPE + Pgp3 group were *C. muridarum* IFU negative in the lower genital tract 20 days post *C. muridarum* challenging (**Tables 1**, **2**), and 100% of mice were negative 26 days post *C. muridarum* challenging. Therefore, GPE assisted Pgp3 to establish better immunoprotection.

**Table 1:** Area under the *C. muridarum* Log10 (IFU)-days post-infection line chart.

	PBS	Pgp3	GPE + Pgp3
Area under the line chart	20.8	14.51	9.03

**Table 2:** Percentage of IFU-positive mice post *C. muridarum* challenging.

	D7	D14	D17	D20	D23	D26	D29	D32	D35
PBS	100	100	100	100	100	100	100	80	0
Pgp3	100	100	100	100	100	80	0	0	0
GPE + Pgp3	100	100	100	60	40	0	0	0	0

**Figure 11:**
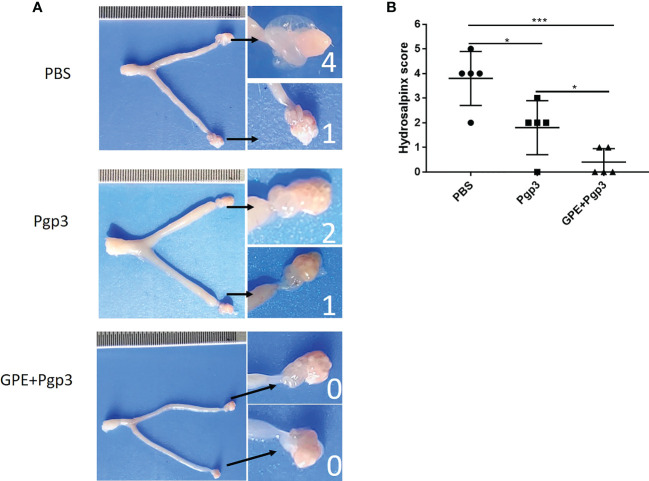
Histopathological changes and hydrosalpinx in genital tract post **(C)** *muradirum* challenging. **(A)** Hydrosalpinx of a representative mouse. The areas covering the oviduct portions were magnified and are shown on the right side of the corresponding images of whole genital tracts. Hydrosalpinx indicated by black arrows and hydrosalpinx severity scores indicated by white numbers; **(B)** Hydrosalpinx score of each group of mice, with the two sides scores added together to obtain the final score of one mouse. Severity of hydrosalpinx listed along the Y-axis and the three different vaccination groups listed along the X-axis. *N* = 5.).

In addition, hydrosalpinx was one of the typical symptoms of chronic inflammation after *C. muridarum* infection. The PBS control displayed the highest cumulative hydrosalpinx score, indicating the most serious hydrosalpinx, with the fallopian tubes in a transparent state and being even larger in size than the ovary (**Figure 11A**). The unilateral hydrosalpinx rate of the Pgp3 group was lower than that of the PBS group, with the former’s cumulative score being nearly half of the latter’s, and the diameters of the fallopian tubes of mice in the Pgp3 group being smaller than those of mice in the PBS group (**Figure 11A**; **Table 3**). The GPE adjuvant further reduced hydrosalpinx, with mice in the GPE + Pgp3 group having no bilateral hydrosalpinx, and the smallest hydrosalpinx scores compared with those in the PBS and Pgp3 groups. In addition, there was no obvious dilation detected in the oviduct tissues of mice in the GPE + Pgp3 group (**Figure 11B**, **Table 3**).

**Table 3:** Hydrosalpinx rate and cumulative score of infected mice.

	PBS	Pgp3	GPE + Pgp3
Bilateral hydrosalpinx	2/5	1/5	0/5
Unilateral hydrosalpinx	5/5	4/5	2/5
Cumulative hydrosalpinx score	19	10	2

**Figure 12:**
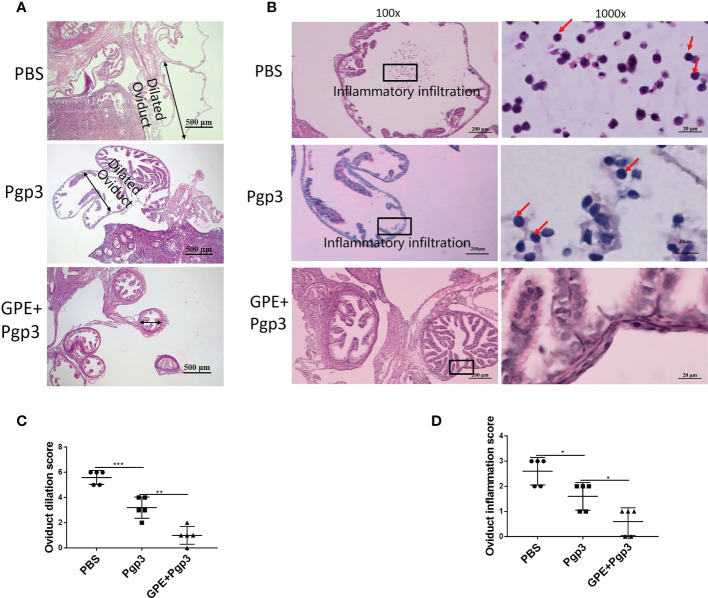
Microscopic observation of oviduct dilation and inflammatory infiltration. The oviduct tissues of vaccinated mice were harvested 60 days post *Chlamydia muridarum* (*C. muridarum*) infection and subjected to H&E staining. **(A)** Representative images of oviduct dilation. Images were magnified ×50; **(B)** inflammatory infiltration of the oviduct with one representative image of a H&E stained oviduct from each group of mice presented in the left. The areas indicated by the black rectangles, covering the oviduct lumen, were magnified and are shown on the right side of the corresponding images, with the inflammatory cells indicated by red arrows. Images were magnified ×100 (left) and ×1,000 (right); **(C)** histograms of oviduct dilation scores; **(D)** oviduct inflammation scores. *n* = 5. H&E, hematoxylin and eosin.

Improvements in reproductive tract pathology were evidence to further evaluate immune protection against *C. muridarum* infection. The histological sections of mice in the PBS group showed that the oviduct layers became thinner, with the oviduct lumina significantly dilated, and that both the plica mucosa and oviductal motile ciliated cells were either reduced or disappeared (**Figure 12A**). Moreover, the highest levels of inflammatory cells and foci were recorded for this group (**Figure 12B**). The Pgp3 vaccination protected the plica mucosa and oviduct dilation, and only several focal inflammations were observed in mice in this group (**Figures 12A–D**). The GPE + Pgp3 treatment further protected the oviduct from *C. muridarum* infection as no obvious dilation was found in its appearance, as revealed by hematoxylin and eosin (H&E) staining. Microscopies of whole reproductive tracts revealed that there were few inflammatory cell infiltrations in mice in this group, and inflammation scores were also significantly reduced compared with the PBS and Pgp3 groups (**Figure 12D**).

**Figure 13:**
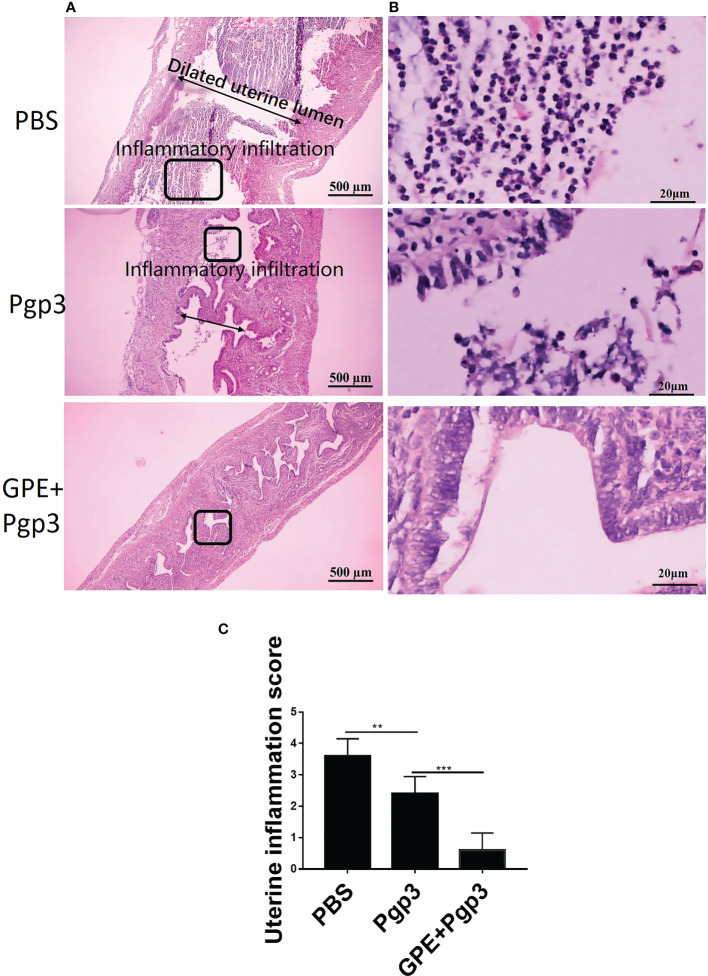
Microscopic observation of uterine horn dilation and inflammatory infiltration. The oviduct tissues of vaccinated mice were harvested 60 days post *Chlamydia muridarum (C. muridarum)* infection and subjected to H&E staining. **(A)** Representative images of the uterine. Images were magnified ×50; **(B)** Inflammatory infiltrated areas as indicated by the rectangles at ×1,000 magnification; **(C)** histograms for uterine inflammation scores. *n* = 5. H&E, hematoxylin and eosin.

Similarly, chronic pathological changes in the uterine horn were evaluated based on the severity of uterine glandular duct dilation and number of inflammatory lesions. The uterine horn tissues of mice in the GPE+Pgp3 group turned out well-defined small lumina and an intact endometrial layer, with tubular structures that extended into the glandular ducts in the endometrial stromata (**Figure 13A**). In contrast, the H&E staining sections from mice injected with PBS showed the most severe pathological changes, because the uterine lumina of mice in the PBS group were infiltrated by inflammatory cells and the glandular ducts appeared dilated, whereas mice in the Pgp3 group had slightly reduced intrauterine dilation and inflammation scores when compared with those in the PBS group (**Figures 13A–C**).

## Discussion

Pickering emulsion formation was associated with various factors. Power input, particle type and proportion, salinity and pH, and water/oil ratio were important influencing factors. The small size and consistent morphology of the emulsion droplets in our work suggested that they might be beneficial for biological application, especially antigen delivery. First, changes in GO concentration were consistent with other studies, such as one investigating GO-benzyl chloride (27) and GO-ionic liquid Pickering emulsions (28), as well as those Pickering emulsions prepared by other solid particles (29). In addition, particle concentration and water/oil ratio had a remarkable influence on the emulsion stability and droplet size because increased GO concentration on the oil−water interface reduced the free energy, made the system more stable, and reduced the droplet size, findings which have been confirmed by nearly all related research (30). A high water/oil ratio resulted in a higher GO concentration, which also reduced the droplet size because the ability of GO to act as a surfactant was strongly favored by squalene, a non-polar and aromatic oil phase (31, 32). Finally, for salinity and pH, some similar research also supported our result that changes in salinity and pH might alter GO’s amphiphilic properties (33), because base-enhanced deprotonation of carboxylic acid groups on the nanosheet edges improved their dispersibility, further affecting droplet stability and size (34, 35). For instance, the droplet size of an ionic liquid-containing Pickering emulsion stabilized by a GO-based surfactant was significantly enlarged with the addition of HCl or NaOH (28). Our optimization of GPE generally followed the Pickering formation and change rule as most previous research, and the GPE droplet with the smallest size was chosen as an adjuvant candidate after weighing the biocompatibility and cost of GPE by minimizing the use of GO and squalene.

Antigen-controlled release is also one of the factors that has an important impact on the immunostimulatory activity of an emulsion adjuvant (8), since rapid release would facilitate not only the direct exposure of antigens to tissue fluids, but also the wide cellular uptake by APCs that had not been sensitized by the antigen or adjuvant, thus leading to compromised efficacy of vaccination (36, 37). Spontaneous antigen adsorption on GO nanosheets attenuated the burst release of antigens, which was beneficial for both the depot effect at the injection site and efficient intracellular vaccine delivery (38). In general, nanoparticles carry cargoes *via* complex interactions. GPE adsorbed protein largely by electrostatic attraction, complexation, and hydrophobic π–π interaction. The strong hydrophobic interaction, especially the π–π interaction between proteins and the GO-based composite, was the main driving force for protein adsorption. For instance, the presence of hydrophobic aromatic groups on the GO surface could favor hydrophobically driven protein adsorption, whereas the presence of epoxide moieties could enable covalent reactions with lysine units. Hydrophobic GPE could even facilitate the delivery of antigens with hydrophobic domains that would otherwise precipitate in a pure aqueous medium (39). Although the efficiency of the electronegative protein was reduced because of the decreased electrostatic attraction between the negatively charged protein and the negatively charged GPE droplet (40), proteins such as BSA or Pgp3 could still be efficiently adsorbed on the GPE droplet interface by strong π–π interaction. In addition to efficient adsorption, it is worth noting that the secondary and tertiary structures of proteins might change in the presence of GO (41). GPE assisted Pgp3 to elicit enhanced immunoprotection against *C. muridarum*, which was based on natural Pgp3’s trimer conformation; the result indicated that GPE might reduce the protein conformation perturbation factors caused by GO.

Emulsion adjuvants are known to induce a local proinflammatory reaction at the site of administration. The recruitment of macrophages is therefore important for innate immune cells after immunization. GPE significantly improved macrophage recruitment at the injection site, which resulted in efficient Pgp3 recognition and presentation because the well-trained macrophages induced rapid protection against infection in mice (42), and the adjuvant further increased the macrophages’ killing ability (43), as our *in vitro* results displayed.

We further focused the adjuvant mechanism of GPE on macrophage cytokine stimulation and polarization *in vitro*. Previous studies showed that TNF-α and IL-6 secretion were up-regulated following GO treatment of macrophages (44, 45). It can be hypothesized that inflammatory factor secretion by GPE stimulation involving TNF-α mediated immune responses was probably largely mediated by GO *via* inducing inflammatory cytokines (46). In fact, macrophage polarization plays a major role in immune stimulation. M1/M2 polarization of macrophages was regulated by surrounding inflammatory mediators after pathogenic infection. Imbalanced polarization of classically activated (M1) and alternatively activated (M2) macrophages was closely associated with pathogen infection and the *in vivo* immune response (47). M1-polarized macrophage hosts were immune protected and were substantially accelerated by prior vaccination with the vaccine and adjuvant (48, 49), whereas those biased on the M2 side helped chronic infection (7). We assessed the percentages of M1 (pro-inflammatory) and M2 (reparative) macrophages through the expression of specific markers CD86 (M1) and CD206 (M2), respectively. The difference in CD206+ was statistically insignificant, confirming that their stimulation for M2 polarization was ineffective *in vitro*.

The relationship between the emulsions and the intensity and type of the immune response is complex (36). The physicochemical properties of the emulsion had a significant influence on the adjuvant and delivery efficacy (50–52). The crucial influencing parameters included membrane fluidity (8), surface charge, and physical size, modulating the biological behaviors of antigens and ultimately providing a collective effect on the immunostimulatory activity (53–55). First, small-sized GPE droplets were generally positively correlated with cellular uptake. GPE droplets sized 0.5–3 μm were efficiently taken up by APCs and drained into the lymph node (56) since particle sizes typically ranging from 0.5 to 5.0 μm are largely taken up through macropinocytosis and dorsal ruffles (57). Our *in vitro* results also indicated that GPE adsorption enhanced Pgp3 uptake efficiency for easy recognition by macrophages. Subsequently, the chemical properties of squalene and GO significantly affected immune stimulation. Squalene was the main component of many approved emulsion adjuvants, and stimulated cellular immunity *via* the recruitment of granulocytes and macrophages and regulated the downstream expression of MHC II and costimulatory molecules (55). For instance, a particulate alum-based Pickering emulsion synthesized using squalene enhanced the antibody titer six-fold and IFN-γ threefold compared with traditional aluminum adjuvant (58). GO also served as a vaccine carrier and showed significant adjuvant activity in activating cellular and humoral immunity (15, 16), because the quasi-2D structure and specific surface area provided numerous antigen adsorption sites and contributed to the slow release, recognition, and presentation of antigen (59). In general, GPE, with a small-sized droplet, and further due to the immunostimulatory activities of GO and squalene, elicited more than 10 times the level of IgG antibody serum and four times the levels of IFN-γ and IL-2 than that of the Pgp3 group, making it the superior adjuvant candidate.

Moreover, in our previous study, traditional emulsions stimulated IgG production rather than cytokine production (3), and the widely approved aluminum adjuvant stimulated only poor cellular immunity. The higher cytokine levels stimulated by GPE indicated efficient cellular immunity, which was a key factor in immunoprotection against *C. trachomatis*. Th1-polarizing cytokines, such as IFN-γ and IL-2 production, could significantly accelerate *C. muridarum* clearance and reduce genital tract damage, which was largely mediated by interferon IFN-γ-producing CD4^+^ T cells. Pgp3 stimulation played a dominant role in Th1 polarizing cytokine and GPE enhanced Pgp3’s immunostimulation (60). In fact, the production of IFN-γ was critical for inhibiting the development of *C. trachomatis*, because IFN-γ was able to enhance the phagocytic capabilities of macrophages, and may also promote the engulfment and elimination of *C. trachomatis* (61). Systemic and mucosal IgG antibody production post immunization, and mucosal sIgA antibody production post *C. muridarum* challenging, together with Th1 type cytokines, were the main reasons for *C. trachomatis* clearance and pathologic change.

## Conclusions

A relatively small GPE with a narrow distribution droplet was prepared by sonication at 163 W for 2 min, with a pH of 2 and low PBS concentration, 1 mg/mL GO, and a water/oil ratio of 10:1. Mice in the GPE group showed the controlled release of antigens *in vitro* and *in vivo*, enhanced cellular uptake, and TNF-α proinflammatory cytokine secretion by macrophages, which were efficiently recruited at the injection site, and also modulated its M1 polarization. When co-vaccinated with Pgp3, GPE stimulated high levels of IgG, IgG1, and IgG2a, successfully enhanced Th1-type cellular immunity by promoting IFN-γ and IL-2 secretion, and, finally, enhanced immunoprotection against *C. muridarum* infection, alleviating inflammation and tissue damage in the genital tract.

## Data availability statement

The original contributions presented in the study are included in the article/**Supplementary Material**. Further inquiries can be directed to the corresponding author.

## Ethics statement

The animal study was reviewed and approved by Animal ethics committee of the University of South China.

## Author contributions

LZ conducted the optimization and characterization of all materials and wrote the original draft. MS and KS performed the animal and *in vitro* experiment. ZL conceived the idea and supervised the project. HC revised the paper. All authors contributed to the article and approved the submitted version.
