# Supplementary material for: Preparation of graphene oxide–stabilized Pickering emulsion adjuvant for Pgp3 recombinant vaccine and enhanced immunoprotection against Chlamydia Trachomatis infection
Source: Front Immunol. 2023 Apr 18;14:1148253. doi: 10.3389/fimmu.2023.1148253 (PMC10152066; doi:10.3389/fimmu.2023.1148253)
Supplement: Supplementary file 1 [file DataSheet_1.doc]

Supplementary Material

**Preparation of Graphene Oxide Stabilized Pickering Emulsion Adjuvant for Pgp3 R[ecombinant](../../../Administrator/AppData/Local/youdao/dict/Application/8.9.3.0/resultui/html/index.html#/javascript:;) Vaccine and** **Enhanced Immunoprotection against *Chlamydia Trachomatis* Infection**

Lanhua Zhao ^1^, Mingyi Shu ^1^, Hongliang Chen ^2^, Keliang Shi ^1^, Zhongyu Li ^1^*

^1^. *Institute of Pathogenic Biology, Hengyang Medical College, Hunan Provincial Key Laboratory for Special Pathogens Prevention and Control, Hunan Province Cooperative Innovation Center for Molecular Target New Drug Study,* *University of South China, Hengyang, 421001, Hunan, People's Republic of China*

*^2^. Laboratory Department of Chenzhou First People's Hospital, Chenzhou，423000, China*

* Correspondence: Zhongyu Li E-mail: lzhy1023@hotmail.com

**S1. Effect of sonication power on GPE**


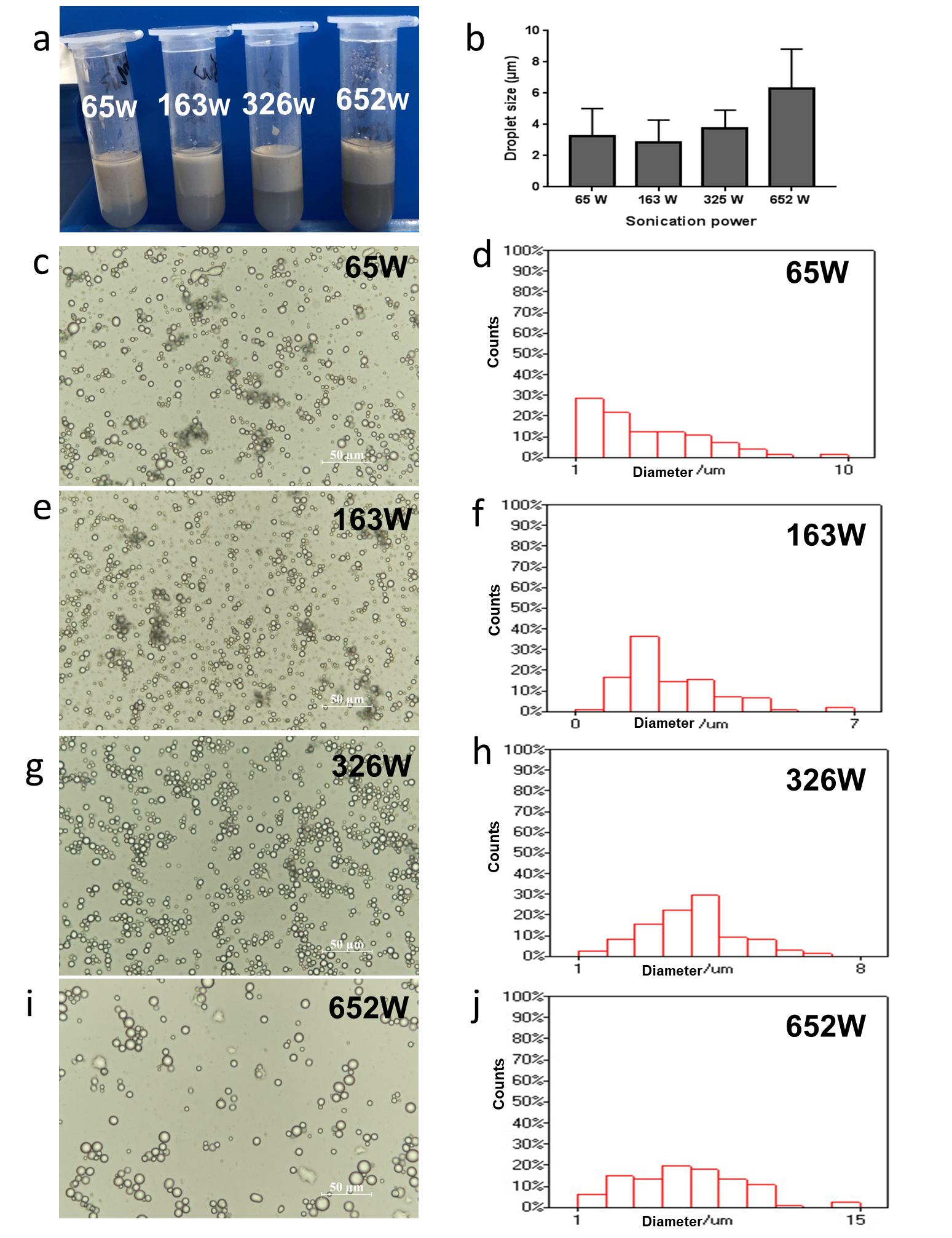


**SUPPLEMENTAL FIGURE 1.** (a) GPEs prepared by different sonication power, (b) the average droplet sizes as a function of sonication power, (c, e, g, i) optical micrographs and (d, f, h, j) droplet size distribution of the GPE prepared by different sonication power (c, d: 65 W; e and f: 163 W; g and h: 326 W; I and j: 652 W).

Preparation conditions: water/oil ratio 10:2, ultrasonacation for 2 min, GO concentration 1 mg/mL, natural salinity and pH condition.


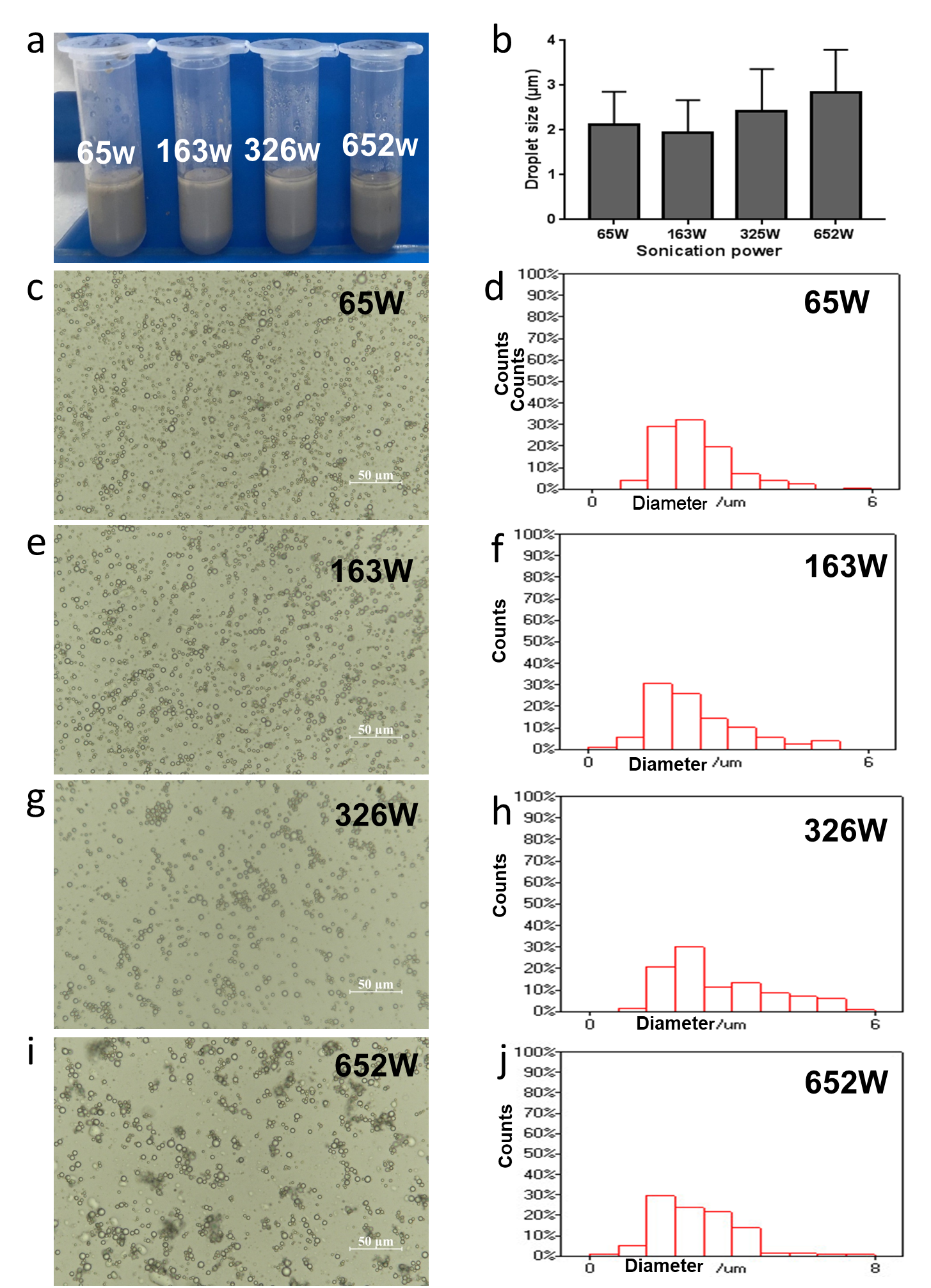


**SUPPLEMENTAL FIGURE 2**. (a) GPEs prepared by different sonication power, (b) the average droplet sizes as a function of sonication power, (c, e, g, i) optical micrographs and (d, f, h, j) droplet size distribution of the GPE prepared by different sonication power.

Preparation conditions as that of Fig.S1 except of GO concentration was 2 mg/mL

**S2. Effect of sonication time on GPE**


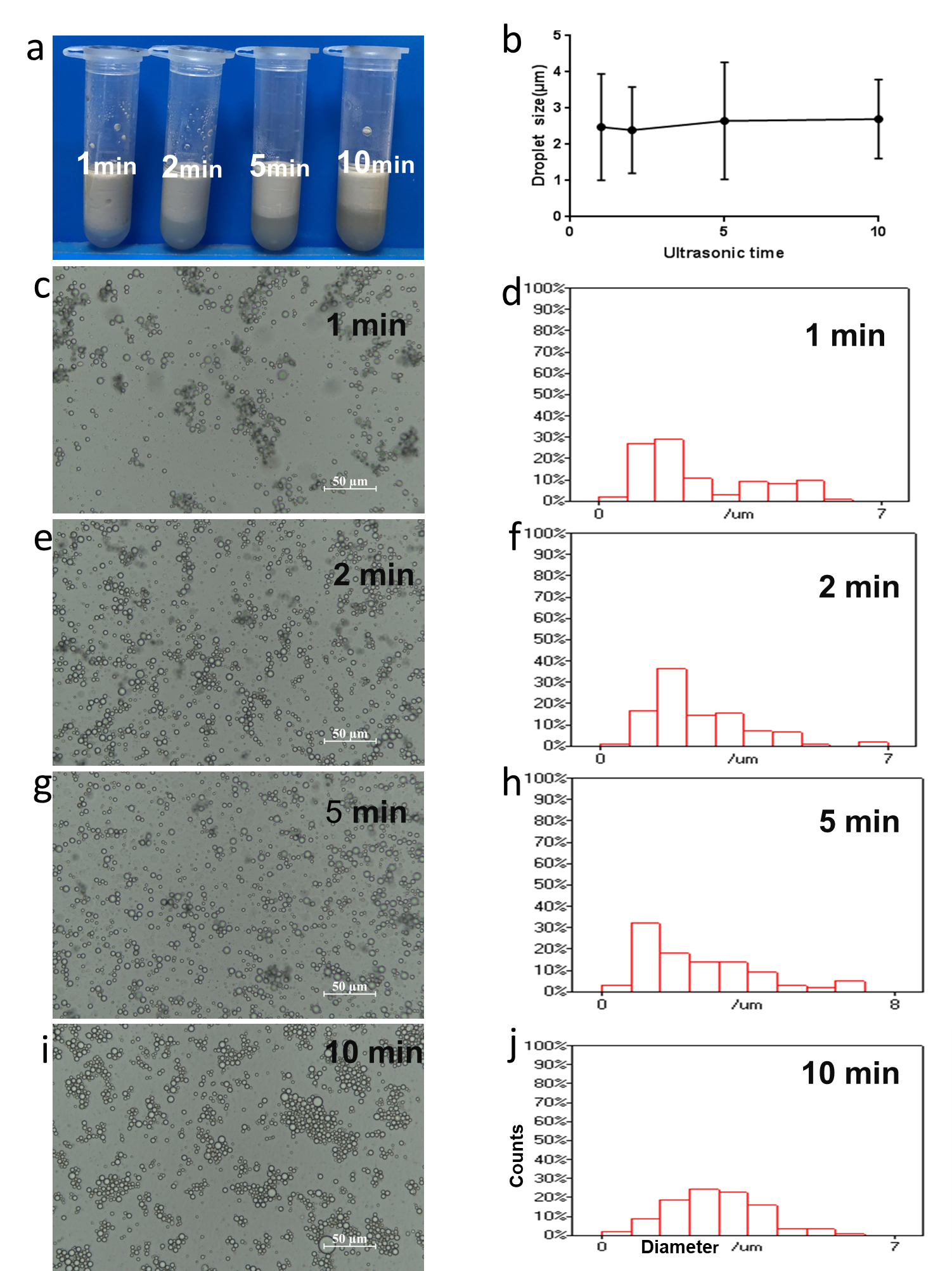


**SUPPLEMENTAL FIGURE 3**. (a) GPEs prepared by different sonication time, (b) The average droplet sizes as a function of sonication time, (c, e, g, i) Optical micrographs and (d, f, h, j) droplet size distribution of the GPE prepared by different sonication time.

Preparation conditions: water/oil ratio 10:2, sonication at 163 W, GO concentration 1 mg/mL, under natural salinity and pH condition.


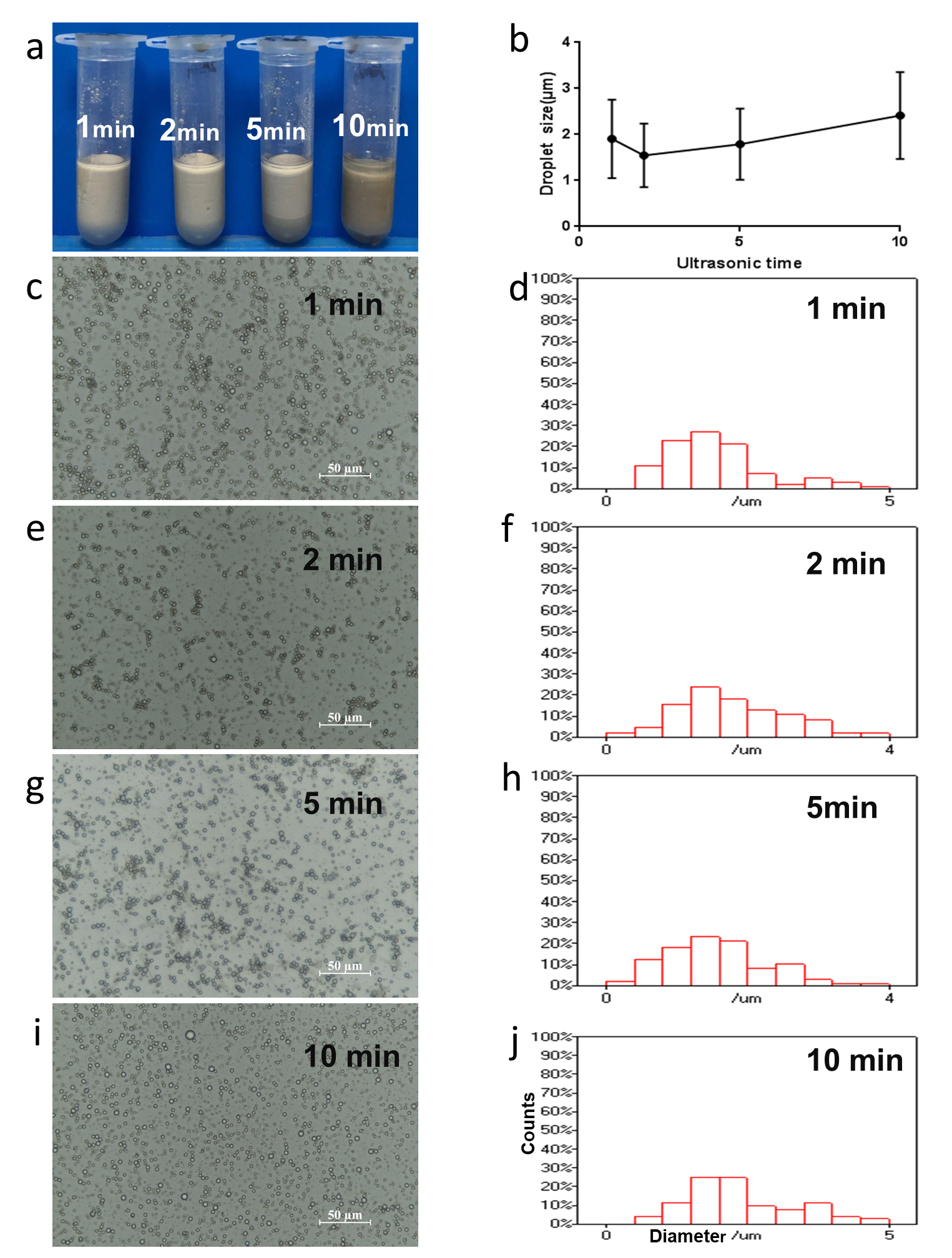


**SUPPLEMENTAL FIGURE 4**. (a) GPEs prepared by different sonication time, (b) The average droplet sizes as a function of sonication time, (c, e, g, i) Optical micrographs and (d, f, h, j) droplet size distribution of the GPE prepared by different sonication time.

Preparation conditions as that of Fig.S3 except of GO concentration was 2 mg/mL

**S3. Effect of pH on GPE**


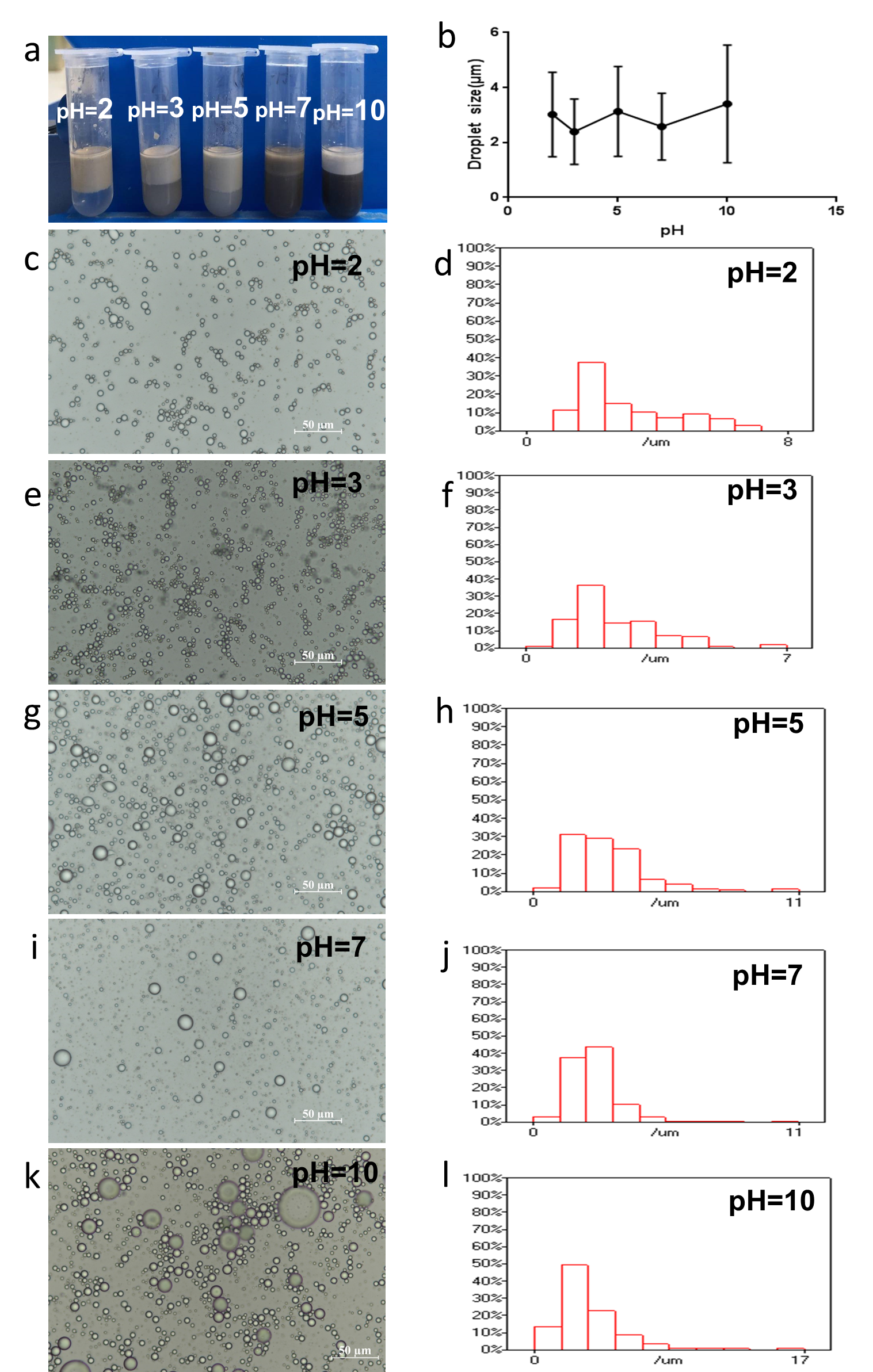


**SUPPLEMENTAL FIGURE 5.** (a) GPEs prepared at different pH, (b) The average droplet sizes as a function of pH, (c, e, g, i) optical micrographs and (d, f, h, j) droplet size distribution of the GPE prepared by different pH. Preparation conditions: water/oil ratio 10:2, GO concentration 1mg/mL, sonication at 163 W for 2 min.


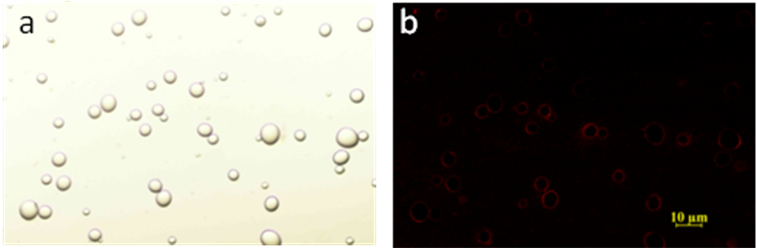


**SUPPLEMENTAL FIGURE 6.** GO dispersion was incubated with rhodamine B overnight and centrifuged at 3,000 r/min several times until no fluorescence was detected in the supernatant to ensure GO location in the Pickering emulsion system. The stained GO dispersion was used to prepare the Pickering emulsion and observation. Optical micrograph of Rhodamine B stained GPE (a. bright field observation; b. dark field observation)

c

d


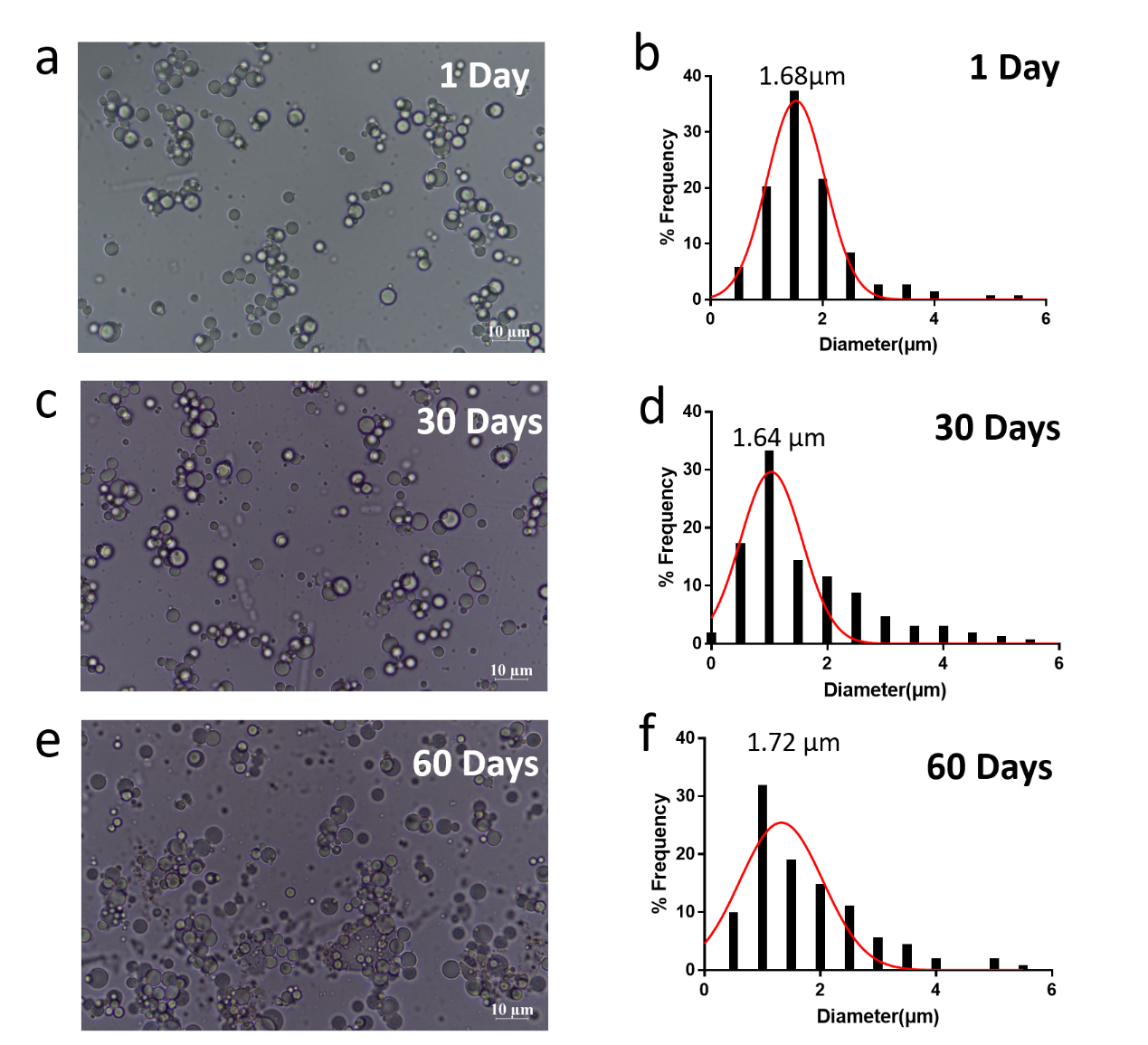


**SUPPLEMENTAL FIGURE 7.** Long-term storage stability of GPE. (a. Optical microscope image GPE placed in room temperature for 1 day; b. Droplet size distribution of image a; c. Optical microscope image GPE placed in room temperature for 30 days; d. Droplet size distribution of image c; e. Optical microscope image GPE placed in room temperature for 60 days; f. Droplet size distribution of image e).

a

b


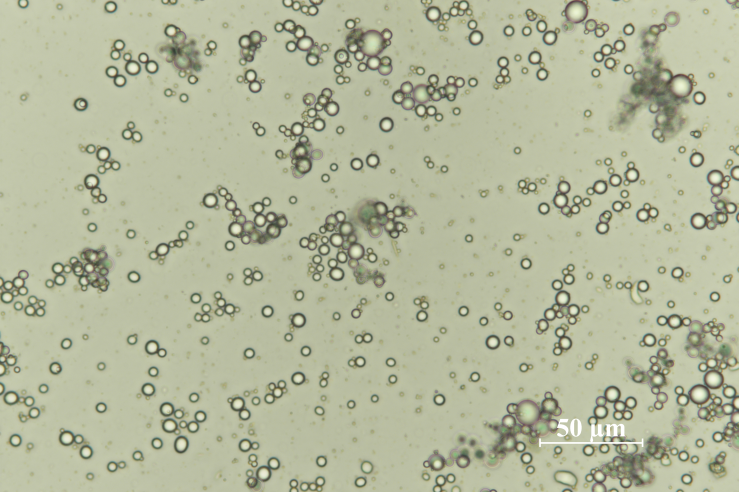


**SUPPLEMENTAL FIGURE 8.** Distribution (a) and Optical micrograph (b) of GPE dealt with 3000 r/min centrifugation and resuspended by PBS.


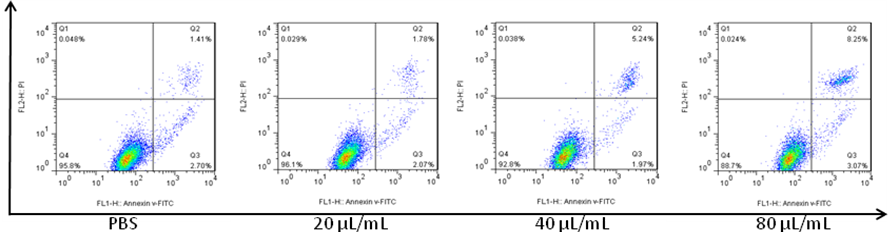


**SUPPLEMENTAL FIGURE 9.** Marcrophage cell apoptosis induced by GPE
